# Lung cancer, comorbidities, and medication: the infernal trio

**DOI:** 10.3389/fphar.2023.1016976

**Published:** 2024-02-21

**Authors:** Hélène Pluchart, Sébastien Chanoine, Denis Moro-Sibilot, Christos Chouaid, Gil Frey, Julie Villa, Bruno Degano, Matteo Giaj Levra, Pierrick Bedouch, Anne-Claire Toffart

**Affiliations:** ^1^ Pôle Pharmacie, Centre Hospitalier Universitaire Grenoble Alpes, La Tronche, France; ^2^ Université Grenoble Alpes, Grenoble, France; ^3^ Université Grenoble Alpes, CNRS, Grenoble INP, TIMC UMR5525, Grenoble, France; ^4^ Institut pour l’Avancée des Biosciences, UGA/INSERM U1209/CNRS 5309, Université Grenoble Alpes, La Tronche, France; ^5^ Service Hospitalier Universitaire de Pneumologie Physiologie, Centre Hospitalier Universitaire Grenoble Alpes, Grenoble, France; ^6^ Service de Pneumologie, Centre Hospitalier Intercommunal de Créteil, Créteil, France; ^7^ Inserm U955, UPEC, IMRB, équipe CEpiA, Créteil France; ^8^ Service de Chirurgie Thoracique, Vasculaire et Endocrinienne, Centre Hospitalier Universitaire Grenoble Alpes, Grenoble, France; ^9^ Service de Radiothérapie, Centre Hospitalier Universitaire Grenoble Alpes, Grenoble, France; ^10^ Laboratoire HP2, INSERM U1042, Université Grenoble Alpes, Grenoble, France

**Keywords:** comorbidities, lung cancer, survival, polypharmacy, drug-drug interaction

## Abstract

Most patients with lung cancer are smokers and are of advanced age. They are therefore at high risk of having age- and lifestyle-related comorbidities. These comorbidities are subject to treatment or even polypharmacy. There is growing evidence of a link between lung cancer, comorbidities and medications. The relationships between these entities are complex. The presence of comorbidities and their treatments influence the time of cancer diagnosis, as well as the diagnostic and treatment strategy. On the other hand, cancer treatment may have an impact on the patient’s comorbidities such as renal failure, pneumonitis or endocrinopathies. This review highlights how some comorbidities may have an impact on lung cancer presentation and may require treatment adjustments. Reciprocal influences between the treatment of comorbidities and anticancer therapy will also be discussed.

## 1 Background

Lung cancer is often diagnosed at an advanced stage of the disease (Jemal et al., 2010; Howlader et al., 2011), at a median age of 70 years (Noone et al., 2015). At this age, 65% of the general population has at least two comorbidities (Divo et al., 2014). The prevalence of comorbidities for lung cancer patients is higher than that of other cancer patients, approximately 50%–70% at diagnosis (Edwards et al., 2014a; Islam et al., 2015). However, little is known about lung cancer in elderly patients with comorbidities because they are excluded from most clinical trials (Hutchins et al., 1999).

On the one hand, comorbidities can have an impact on cancer survival (Islam et al., 2015; Leduc et al., 2017; Morishima et al., 2019) by influencing the therapeutic strategy. They could be responsible to an alteration of the general condition or a worse tolerance of anticancer treatments. In addition anticancer treatments can worsen or induce comorbidities (Laskin et al., 2012; Perazella, 2012; Haanen et al., 2017; McGowan et al., 2017; Hanania et al., 2019).

On the other hand, comorbidities generally imply the addition of medication to the symptomatic treatment intrinsically related to lung cancer. Such medication is likely to have consequences on drug-drug interactions or non-adherence or be responsible for adverse drug reactions (Scripture and Figg, 2006a; Popa et al., 2014a). Such multiple prescriptions can lead to polypharmacy (PP) (generally defined as a threshold of five medications) (Masnoon et al., 2017) and the administration of potentially inappropriate medications, which has been described for cancer patients, in particular, the elderly (Lees and Chan, 2011; LeBlanc et al., 2015; Nightingale et al., 2015).

**FIGURE 1:**
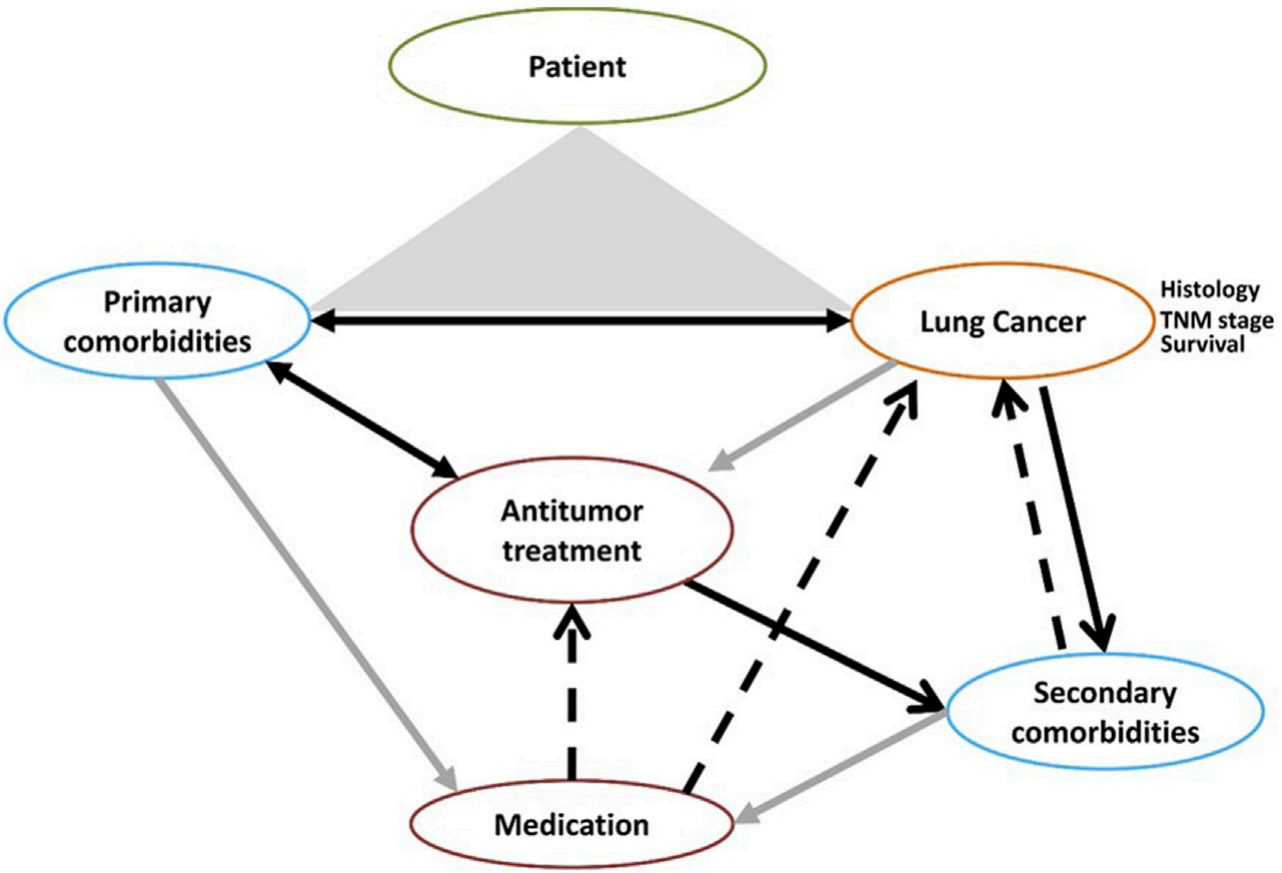
Lung, cancer, medication, and comobidities: an infernal trio.

This paradigm concerns all cancers, and we have chosen to focus on lung cancer. Very few data are available specifically on lung cancer. That is why we also presented publications (study or review) on all solid tumors. Although lung cancer population can be different from other cancer types, drug pharmacokinetics and pharmacodynamics are similar. Here, we propose a narrative review on the existing literature concerning the impact of both comorbidities and medication on lung cancer patients, as well as the consequences of anticancer treatments on comorbidities (Figure 1).

## 2 Lung cancer and comorbidities

Comorbidity was first defined as the existence of any distinct additional clinical entity by Alvan Feinstein in 1970 (Feinstein, 1970). Comorbidity can be scored by several means. The most used in lung cancer is the Charlson Comorbidity Index (CCI), which assesses the impact of comorbidities (Charlson et al., 1987). There are several scores to describe the impact of comorbidities (Sarfati, 2012). More than half (50%–70%) of lung cancer patients have comorbidities at diagnosis (Edwards et al., 2014b; Islam et al., 2015). In 90% of cases (Barbone et al., 1997), lung cancer is associated with tobacco consumption, which can lead to lung injury, cardiovascular diseases, and diabetes mellitus (Chang, 2012; Goldkorn et al., 2014; Messner and Bernhard, 2014). Most lung cancer patients are over 65 years of age (Noone et al., 2015). Ageing can be associated with a decline in renal function and cardiovascular and metabolic disorders (Scheen, 2005; Halter et al., 2014; Mallappallil et al., 2014). The most frequent comorbidity of lung cancer patients is chronic obstructive pulmonary disease (COPD) (30%–50% of lung cancer patients), followed by diabetes (15%), congestive heart failure (12%), peripheral vascular disease (7%–8%), cerebrovascular disease (7%), and renal disease (from 2% to 6%) (Edwards et al., 2014b; Islam et al., 2015).

We first discuss the impact of comorbidities on lung cancer and the impact of lung cancer on comorbidities.

### 2.1 Impact of tobacco exposure and comorbidities on lung cancer features and treatments

Certain comorbidities taken separately are reported as independent poor prognostic factors for the survival of lung cancer patients (Leduc et al., 2017), such as COPD, tuberculosis, interstitial lung disease, cardiovascular diseases, and renal insufficiency (Table 1). Obesity is described as a favorable prognostic factor, within the framework of “obesity paradox” described in lung cancer (Li et al., 2016; Petrelli et al., 2021). While low body mass index is associated with lower survival, patients with obesity have improved survival.

**TABLE 1:** Impact of tobacco exposure and comorbidities on lung cancer features and treatments.

Cancer characteristics or treatment		Comorbidities or risk factor exposure				
		Tobacco use	Chronic pulmonary diseases (or COPD)	Cardiovascular history	Diabetes mellitus	Increasing number or severity of comorbidities
**Cancer features**	**Histology**	**high smoking intensity**: ↗ the risk of SCLC (11-fold), 18-fold of SqCC (18-fold), and of ADC (4-fold) Khuder (2001)	**Tuberculosis** ↗ (2-fold) the risk of ADC Liang et al. (2009)			
			**COPD** ↗ (4-fold) the risk of SqCC Papi et al. (2004); de Torres et al. (2011)			
			**Asthma** ↗ (2-fold) the risk of SqCC or SCLC Boffetta et al. (2002); Rosenberger et al. (2012)			
	**Stage at diagnosis**	**Screening in smokers**: earlier stage at diagnosis and ↗ survival Aberle et al. (2011); Leroy et al. (2017); de Koning et al. (2020)				Trend towards earlier stage at diagnosis Pagano et al. (2010); Lüchtenborg et al. (2012); Wang et al. (2012)
**Cancer treatments**	**Surgery**		**COPD**: sub-lobar surgery preferred to lobectomy Eguchi et al. (2017)	Preferentially sub-lobar surgery rather than lobectomy Eguchi et al. (2017)		Less proposed Lüchtenborg et al. (2012)
						↗ complications Birim et al. (2003)
						↘ survival Powell et al. (2013)
						Video-assisted thoracoscopy preferred to open surgery Jawitz et al. (2017)
	**Radiotherapy**	↗ radiation pneumonitis Monson et al. (1998)		↗ radiation pneumonitis Nalbantov et al. (2013)		Preferred to surgery in early stages Nilsson et al. (2017)
	**Chemotherapy**			↗ cisplatin nephropathy Mathe et al. (2011)	↗ cisplatin nephropathy Mathe et al. (2011)	Less prescribed Tammemagi et al. (2004); Grønberg et al. (2010); Jawitz et al. (2017)
						↗ complications Grønberg et al. (2010)
	**Immune oncology treatments**		**Interstitial lung disease** ↗ incidence or immune-related pneumonitis			Less prescribed, particularly if concomitant auto-immune disease Champiat et al. (2016)

ADC, adenocarcinoma; COPD, chronic obstructive pulmonary disease; SCLC, small cell lung cancer; SqCC, squamous cell carcinoma.

Furthermore, comorbidities could induce carcinogenesis, such as COPD (de Torres et al., 2011), infections (Kuper et al., 2000; Yu et al., 2011), diabetes mellitus (although still debated (Sciacca et al., 2013)) and obstructive sleep apnea through hypoxia (Gozal et al., 2015; Justeau et al., 2020). Several mechanisms have been proposed, mostly oxidative damage and chronic inflammation. Comorbidities can affect various lung cancer characteristics such as histology, stage at diagnosis, and anticancer treatment.

#### 2.1.1 Impact of tobacco exposure and comorbidities on histology

COPD increases the frequency of squamous cell carcinoma by four fold (Papi et al., 2004; de Torres et al., 2011), whereas asthma increases the frequency of this histological subtype or of small-cell lung cancer (SCLC) by two fold (Boffetta et al., 2002; Rosenberger et al., 2012). The mechanism is still unclear but may involve an altered inflammatory response and fibrogenesis. Lung cancer patients who had prior tuberculosis show a higher frequency of adenocarcinoma (approximately two-fold higher) (Liang et al., 2009), probably also due to fibrosis and inflammation (Leduc et al., 2017).

Tobacco smoke has been shown to be strongly associated with SCLC and squamous cell carcinoma (proximal localization) (Khuder, 2001). The relationship between histology and the size of inhaled smoke particles was already described in 1989, supporting the hypothesis that the biggest particles settle on cells of the epidermal bronchi and the smallest in peripheral tissue near glandular cells (Yang et al., 1989). In addition, different molecular profiles have been described between smokers and never-smokers with non-small-cell lung cancer (NSCLC). Barlesi et al., 2016 reported that nearly 44% of patients who were never smokers had a mutation in the epidermal growth factor receptor (EGFR) gene (vs. 11% in the overall population). The carcinogens contained in tobacco smoke induce, instead, loss-of-function mutations in tumor suppressor genes, such as p53 (Couraud et al., 2012).

#### 2.1.2 Impact of tobacco exposure and comorbidities on stage at diagnosis

Several studies have investigated how comorbidities affect the stage at diagnosis of lung cancer. However, most showed only a trend towards earlier stages for patients with more comorbidities (Pagano et al., 2010; Lüchtenborg et al., 2012; Wang et al., 2012). These findings may be due to bias related to higher level of medical monitoring in this population. For 402 patients with advanced NSCLC, a significantly higher proportion of stage IIIb than stage IV was observed at diagnosis for patients with severe comorbidities (Grønberg et al., 2010). Dalton et al. reported similar results, showing that a higher CCI correlated with an earlier stage at diagnosis (Dalton et al., 2015). However, these findings are still debated (Ahn et al., 2013; Sarfati et al., 2016).

Lung cancer screening of smokers or COPD patients with emphysema was investigated in the NELSON, NLST, and AIR trials (Aberle et al., 2011; Leroy et al., 2017; de Koning et al., 2020). These studies aimed to diagnose lung cancer at an early stage, when they are eligible for radical treatment that could improve patient survival. For example, lung cancer-specific mortality (primary outcome) was significantly lower in the screening group in the NELSON study (de Koning et al., 2020).

#### 2.1.3 Impact of comorbidities on treatment strategy

##### 2.1.3.1 Surgery

Studies clearly show that surgery is preferred for patients with no or few comorbidities (Cykert et al., 2010; Lüchtenborg et al., 2012). For example, in the Swedish Lung Cancer Data Base (Nilsson et al., 2017), surgery was performed less for patients in early stages of NSCLC with a CCI ≥3 than those without a comorbidity. In addition, the number of comorbidities has been shown to be associated with morbi-mortality for surgically treated NSCLC patients. A CCI score of 3–4 was predictive of major complications (Birim et al., 2003) and a CCI of 2–4 with higher 90-day mortality after surgery (Powell et al., 2013). In the European Respiratory Society and European Society of Thoracic Surgery guidelines, indications for surgery depend on the morbi-mortality risk, which takes into account comorbidities (Brunelli et al., 2009; Brunelli et al., 2013). Patients can be considered to have a low (<1%), moderate, or high (>10%) risk of mortality, depending on their respiratory and cardio-vascular status. The French Society of Thoracic and Cardio-Vascular Surgery developed a risk model to predict in-hospital mortality for patients undergoing lung surgery. The most frequent comorbidities were evaluated: COPD, arterial hypertension, smoking, heart disease, peripheral vascular disease, history of cancer, and diabetes mellitus (Falcoz et al., 2007). Having at least three comorbid conditions was associated with lower in-hospital survival. The revised cardiac risk index is a widely used tool for cardiac risk stratification before major lung resection (Brunel et al., 2010). It relies on four variables that were identified to be predictive risk factors of major cardiac morbidity (cerebrovascular disease, cardiac ischemia, renal disease, and pneumonectomy) and allows the selection of patients who need further cardiac testing. The burden of comorbidities also has an impact on the choice of surgical strategy. Patients with COPD, a history of cardiovascular disease, or a CCI ≥2 preferentially benefit from sub-lobar surgery rather than lobectomy (Eguchi et al., 2017). For patients with few comorbidities, lobectomy is preferentially offered over sub-lobar resection, because it is associated with higher overall survival (Boyer et al., 2017). Video-assisted thoracoscopic surgery is preferred to open surgery for patients with severe comorbidities (Jawitz et al., 2017).

##### 2.1.3.2 Radiotherapy

In early stages, stereotactic radiotherapy is more likely to be proposed for patients with severe comorbidities (CCI ≥3) (Nilsson et al., 2017). The decision to attempt curative radiotherapy increases with the CCI. However, no impact of the CCI on survival after radiotherapy has been reported (Mellemgaard et al., 2015). Radiation-induced lung injury is a common complication of radiotherapy. In 1998, Monson et al. found that pre-existing comorbid lung disease increases the risk of radiation pneumonitis (Monson et al., 1998) and that tobacco consumption is responsible for more extensive lung injury. Cardiac comorbidity has been found to be associated with a higher risk of radiation-induced lung injury than for patients with no prior cardiac comorbidity (Nalbantov et al., 2013). However, the impact of these comorbidities on radiation-induced lung injury is also still debated (Dreyer et al., 2018).

##### 2.1.3.3 Pharmacological treatment

In terms of chemotherapy-related toxicity, the choice of molecule should be very carefully made to not worsen patients‘ comorbidities. The number of comorbidities has an impact on pharmacological treatment (Lee et al., 2011) and chemotherapy in NSCLC is less prescribed as the number of comorbidities increases (Tammemagi et al., 2004; Grønberg et al., 2010; Jawitz et al., 2017). Patients with a high comorbidity score are more likely to have complications after chemotherapy. NSCLC patients with severe comorbidities have been shown to be more likely to have a higher frequency of thrombocytopenia, febrile neutropenia, or death after neutropenic infections than those without comorbidity (Grønberg et al., 2010). Another study which compared pemetrexed/carboplatin *versus* gemcitabine/carboplatin as first line therapy in stage IIIB/IV NSCLC patients reported that patients with comorbidities have a higher risk of developing grade 3 to 5 gastro-intestinal, cardiovascular, and infectious adverse events, rash, and nausea (Asmis et al., 2008). In addition, cardiovascular disease and diabetes increase the risk of cisplatin-induced nephropathy (Mathe et al., 2011).

Immune oncology treatments, such as immune-checkpoint inhibitors that target programmed cell death (PD)-1, PD-ligand (PD-L) 1, or cytotoxic T-lymphocyte-associated protein 4 (CTLA-4) are routinely used in lung cancer. Given the protective role of PD-(L)1 signaling in immune tolerance (Riella et al., 2012), auto-immune diseases can appear or worsen with PD-(L)1 inhibitors. It is very important to identify the risk of dysimmunity before prescribing immune-checkpoint inhibitors (Champiat et al., 2016). Patients with auto-immune diseases have been excluded from phase 3 clinical trials testing immune oncology therapies. Therefore, prescribing this therapeutic class in the context of a pre-existing auto-immune disease can only be considered after multidisciplinary consultation. It has been reported that preexisting interstitial lung disease is associated with higher incidence of immune-related pneumonitis (Gomatou et al., 2020). The indication of immune-checkpoint inhibitors in these patients should be discussed in a multidisciplinary setting.

While some cytotoxic drugs prescriptions such as platin salts are capped if body surface area is superior to 2 square meters, the question of obesity impact on flat doses for immune oncology treatments arises. In obesity, blood volume increases but less than proportional with the change in body weight. Given that monoclonal antibodies distribute only in the blood plasma and extracellular fluids, for some of them, body weight does not have a significant impact on the volume of distribution. Based on pharmacokinetics, flat doses can be proposed in obese population (Hendrikx et al., 2017), as described in the updated ASCO guidelines (review of 6 studies that included overweight or obese patients with cancer) (Griggs et al., 2021). More interestingly, in some studies obese patients receiving PD-1 inhibitors show better survival than nonobese patients, this may due to increased PD-1 expression through leptin higher levels (Woodall et al., 2020).

### 2.2 Impact of lung cancer treatment on comorbidities

After lung cancer treatment, it is important to distinguish temporary side effects from lasting comorbidities (Table 2). This section will focus on the effect of lung cancer treatment in the exacerbation of pre-existing primary comorbidities or those that lead to irreversible secondary comorbidities.

**TABLE 2:** Impact of lung cancer treatment on comorbidities.

Comorbidities	Lung cancer treatment					
	Surgery	Radiotherapy	Chemotherapy	Anti-angiogenic therapy	Tyrosine kinase inhibitors	Immune oncology treatments
**Cardiovascular comorbidities**	Myocardial infarction, atrial fibrillation Steéphan et al. (2000); Brioude et al. (2019)	Pericarditis, acute coronary syndrome, rhythm and conduction disorders, heart failure Hardy et al. (2010); Wang et al. (2017)	**Anthracyclines:** Pericarditis, acute coronary syndrome, heart failure Pai and Nahata (2000); McGowan et al.(2017); Zarifa et al. (2019)	Hypertension Laskin et al. (2012)	**ALK TKI:** QT prolongation, bradycardia, thrombotic events, hypertension Camidge et al. (2018); Camidge et al. (2019); Hou et al. (2019)	Myocarditis, pericarditis, Takotsubo syndrome, acute coronary syndrome, arrythmias Michel et al. (2019); Zarifa et al. (2019)
			**Cisplatin:** arrhythmias and cardiomyopathy Pai and Nahata (2000); Zarifa et al. (2019)		**Osimertinib (EGFR TKI)**: cardiac failure, atrial fibrillation, QT prolongation Anand et al. (2019)	
			**Anti-microtubule agents:** heart failure, acute coronary syndrome, bradycardia, hypotension Pai and Nahata (2000)			
			**Vinka-alkaloids:** Arrhythmias, acute coronary syndrome Pai and Nahata (2000)			
**Venous and arterial thromboembolism**			**Cisplatin** Lee et al. (2015)	**Bevacizumab** Nalluri et al. (2008); Schutz et al. (2011)		Pneumonitis Haanen et al. (2017)
**Chest diseases**	Acute exacerbation of interstitial diseases, phrenic nerve paralysis Sugiura et al. (2012); Brioude et al. (2019)	Radiation pneumonitis potentially complicated by fibrosis Hanania et al. (2019)			Interstitial lung disease Créquit et al. (2015); Hou et al. (2019)	
**Renal failure**			**Cisplatin**, **pemetrexed**, **gemcitabine** Perazella (2012); Toffart et al. (2014); Izzedine et al. (2006)	**Bevacizumab** Laskin et al. (2012); Perazella (2012); Toffart et al. (2014)		
**Endocrinopathies**						Hypothyroidism Haanen et al. (2017); Iglesias (2018)
						Hypophysitis Haanen et al. (2017)
						Type 1 diabetes mellitus Iglesias (2018)

ALK, anaplastic lymphoma kinase; EGFR, epithelial growth factor receptor; TKI, tyrosine kinase inhibitor.

#### 2.2.1 Impact of surgery on comorbidities

Resection of lung cancer can have a consequence on pre-existing comorbidity. Indeed, reduction of lung parenchyma and altered pulmonary function can provoke acute exacerbation of interstitial pneumonia or phrenic nerve paralysis, leading to long-term decreased pulmonary function (Sugiura et al., 2012). Cardiovascular complications in patients with and without risk factors are also frequent such as myocardial infarction, atrial fibrillation can require antiplatelets or anticoagulants prescription (Steéphan et al., 2000; Brioude et al., 2019). Principal risk factors identified for postoperative morbidity include decreased forced expiratory volume in one second, smoking, age and pre-existing cardiovascular disease (Myrdal et al., 2001; Alloubi et al., 2010; Lugg et al., 2015).

#### 2.2.2 Impact of radiotherapy on comorbidities

In the 6 months following lung irradiation, irreversible radiation pneumonitis appears in up to 25% of cases, potentially complicated by fibrosis (1 year after) (Hanania et al., 2019). Glucocorticoids can be proposed for radiation pneumonitis, whereas patients with radiation pulmonary fibrosis should be given supportive care (Hanania et al., 2019).

Cardiac toxicity has also been described with radiotherapy: effusion, unstable angina, myocardial infarction, and arrhythmias (Wang et al., 2017). In addition, chemoradiation has been reported to be an independent risk factor of conduction disorders, cardiac dysfunction, and heart failure (Hardy et al., 2010).

#### 2.2.3 Impact of oral targeted therapies on comorbidities

EGFR and anaplastic lymphoma kinase (ALK) tyrosine kinase inhibitors (TKIs), prescribed in stage IV NSCLC, are less responsible for chronic adverse events. The main adverse events of ALK TKIs are cardiovascular effects: QT prolongation (Camidge et al., 2019), bradycardia (Camidge et al., 2018), thrombotic events (Hou et al., 2019), or hypertension (Camidge et al., 2018). Osimertinib (EGFR TKI) can also induce cardiac failure, atrial fibrillation, and QT prolongation (Anand et al., 2019). A common toxicity of all TKIs is interstitial lung disease (Hou et al., 2019), which may be a hypersensitivity pneumonitis (Créquit et al., 2015).

#### 2.2.4 Impact of chemotherapy, anti-angiogenic therapies and immune checkpoint inhibitors on comorbidities

These toxicities are not exclusive to lung cancer. They are summarized in Table 2 (references (Pai and Nahata, 2000; Nalluri et al., 2008; Schutz et al., 2011; Laskin et al., 2012; Perazella, 2012; Toffart et al., 2014; Izzedine et al., 2006; Lee et al., 2015; Haanen et al., 2017; McGowan et al., 2017; Iglesias, 2018; Michel et al., 2019; Zarifa et al., 2019)).

## 3 Lung cancer and comorbidity-related medication

### 3.1 Impact of comorbidity-related medications on lung cancer diagnosis

Performance of pulmonary diagnostic procedures (for example, bronchial biopsies, transthoracic needle biopsy) can lead to complications such as minimal or severe bleeding (Facciolongo et al., 2016). Concomitant use of antiplatelets or anticoagulant agents for cardiovascular comorbidities may increase this risk. The physician has to assess the risk of bleeding according to patient risk factor and adapt procedures and/or comorbidity-related medications to enable diagnosis to be carried out in safety. According to the level of procedure bleeding risk and the level of comorbidity severity, anticoagulants or antiplatelets are either stopped before the procedure or maintained according to guidelines, depending on diagnostic procedure (Veitch et al., 2016; Pathak et al., 2017).

### 3.2 Impact of comorbidity-related medications on lung cancer features

Comorbidities often lead to the prescription of medication, whereas support treatment is often proposed to counteract the side effects of anticancer treatment. This “prescribing cascade” increases the number of prescribed medications (Gurwitz, 2004). Consequently, lung cancer patients are at a high risk of iatrogenic drug problems, such as drug-drug interactions, adverse drug reactions, or nonadherence (Goh et al., 2018). Medication can include both prescribed drugs and over-the-counter medication, including complementary and alternative medicines (CAM) (Lees and Chan, 2011). Of note, there are discrepancies in the definition of PP (Bushardt et al., 2008). Several studies have defined PP as two to nine medications (Shah and Hajjar, 2012; Turner et al., 2016), whereas others in the geriatric oncology population have used the threshold of five to six (Gnjidic et al., 2012; Nightingale et al., 2015; Sharma et al., 2016). Medication and PP in elderly cancer patients has been widely described in many studies (Lees and Chan, 2011; Maggiore et al., 2014; LeBlanc et al., 2015; Alkan et al., 2016; Lund et al., 2018). Nightingale et al. (Nightingale et al., 2015) reported a mean number of medications of 9, a prevalence of PP of 41%, and excessive PP of 43% (defined as more than 10 medications) in elderly cancer patients.

**FIGURE 2:**
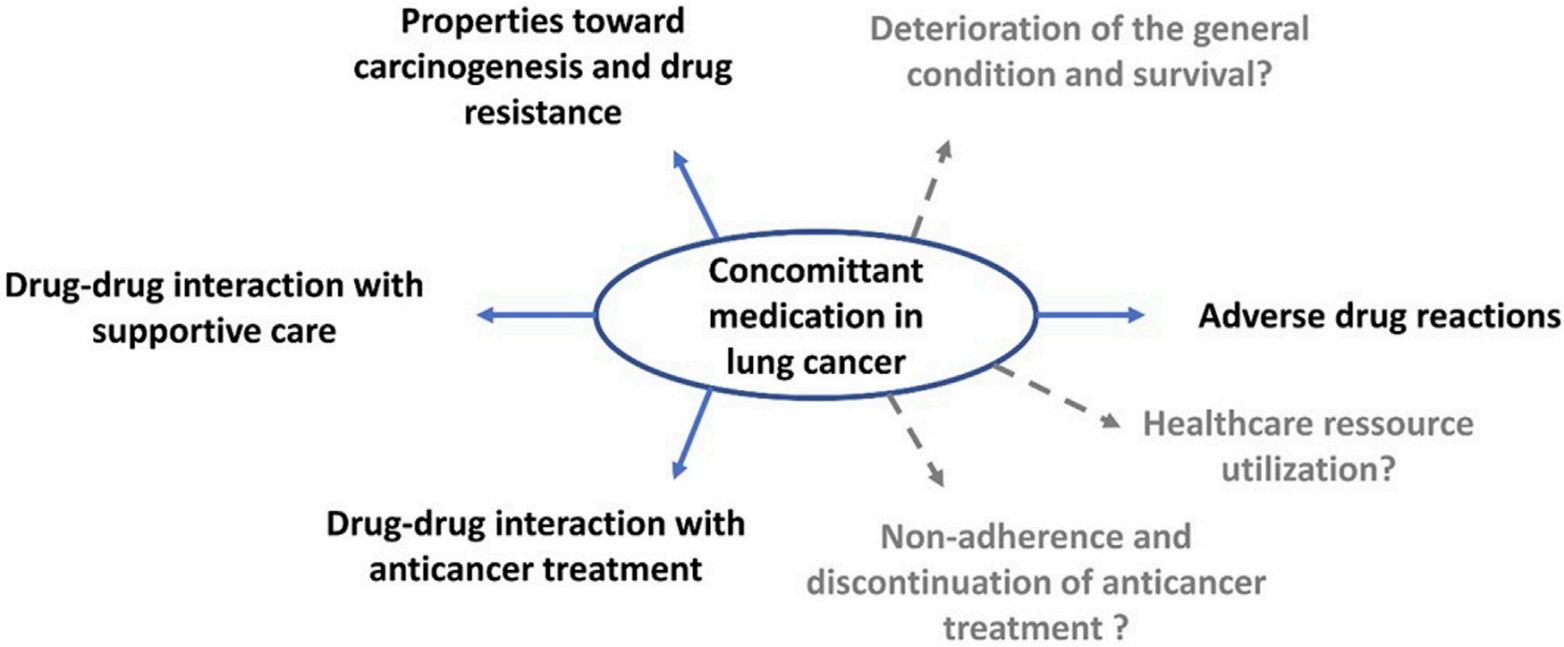
Consequences of concomitant medications in lung cancer patients.

In the following section, we focus on the impact of medications for comorbidities in lung cancer and anticancer treatment, although some of data are not well described in the literature and were deduced from hypotheses based on physiopathology (Figure 2).

#### 3.2.1 Impact of comorbidity-related medications on cancer history

Certain molecules used for the treatment of comorbidities have shown properties towards carcinogenesis or drug resistance.

It appears that AMP-activated kinase, a metformin target, is associated with the tumor suppressor TET2 (Wu et al., 2018). In a recent meta-analysis, metformin showed a possible positive effect in advanced NSCLC, both on overall and progression-free survival (Zhang et al., 2018). The tendency of a favorable effect of statins in cancer has also been described (Han et al., 2011; Nielsen et al., 2012; Cardwell et al., 2015; Lin et al., 2016), although the results have not been consistent between studies (Seckl et al., 2017). There are several possible explanations for such an effect of statins (mevalonate pathway or the inhibition of cholesterol synthesis). Decreased activity of cyclin D1, cyclin-dependent kinase, and metallopeptidase 9 (involved in angiogenesis) by simvastatin would arrest the cell cycle and reduce metastatic potential (Yu et al., 2013). Even more surprisingly, simvastatin appears to reverse the resistance of tumors harboring the T790 M mutation of the EGFR in NSCLC cell lines (Hwang et al., 2014).

Non-steroidal anti-inflammatory drugs may play a positive role in carcinogenesis, the induction of apoptosis, and angiogenesis (Gurpinar et al., 2014). For many years, the literature has suggested that aspirin may have a protective role against cancer, more specifically in colorectal cancer (Garcia-Albeniz and Chan, 2011) and even lung cancer (Muscat et al., 2003).

Lung cancer resistance is caused by various mechanisms (Shanker et al., 2010), such as P-glycoprotein (P-gp), a protein highly expressed in multi-drug resistant lung cancer cells (Cole et al., 1992). P-gp is a drug efflux pump and can decrease antitumor drug concentrations. Verapamil, a calcium channel blocker that inhibits P-gp, may improve the survival of patients being treated with chemotherapy (Millward et al., 1993).

#### 3.2.2 Impact of comorbidity-related medications on drug-drug interactions with anticancer treatment

PP is a risk factor for drug-drug interactions, which can have consequences on the patient, the cancer treatment, and the lung cancer prognosis. Given the narrow therapeutic index of cytotoxic antitumor treatment, it can lead to higher toxicity or therapeutic failure. Drug-drug interactions with antitumor treatment can be pharmacokinetic or involve pharmacodynamic interactions (Scripture and Figg, 2006b). PP, lung cancer diagnosis, and inpatient status are associated with severe drug-drug interactions (Alkan et al., 2016). Potential drug-drug interactions are common among cancer patients and most often involve medications related to comorbid conditions (Riechelmann et al., 2007).

A drug-drug interaction with concomitant medication was detected as a potential problem in 32% of cases in a study of 112 cancer patients, of whom 103 were taking medication for a comorbidity, with a mean number of five medications (Puts et al., 2009). A larger study also reported a frequency of 35% of severe drug-drug interactions among patients (32% of PP), which required considering modification of their therapy or avoidance of the combination. Such potential drug-drug interactions have often been identified with chemotherapy, 14% with a potentially severe impact (Popa et al., 2014b). In a large cohort of 7237 lung cancer patients with stage I/II disease, the most frequent and serious potential drug-drug interactions observed were warfarin associated with etoposide (14%) or gemcitabine (15%). Among the 302 patients treated with cisplatin, 34 (11%) had a concomitant furosemide prescription, which is considered to be a serious potential drug-drug interaction (Lund et al., 2018).

It is well established that TKIs can interact with treatments affecting cytochrome P450 (Song et al., 2011) or with antiacids (Duong and Leung, 2011). A recent review focused on the cardiovascular system and detailed all drug-drug interactions involving cancer treatment and cardiovascular drugs (Akbulut and Urun, 2020). Drug-drug interactions can occur between TKIs and drugs that prolong the QT interval, calcium-channel blockers, diuretics, and anticoagulants. Absorption of the EFGR TKI can be influenced by acid-reducing agents (with a reduced area under the curve) (Peters et al., 2014). A study recently reported that 507 NSCLC patients being concomitantly treated with erlotinib and acid-suppression drugs showed significantly lower progression-free and overall survival (Chu et al., 2015). The same results were found with the concomitant use of erlotinib (Sharma et al., 2019) or gefitinib (Fang et al., 2019) and proton-pump inhibitors, with decreased overall survival. TKIs can be also be involved in metabolism-based interactions through cytochrome P450 activity (Peters et al., 2014).

In recent years, question of gut and lung microbiome role as driver of immune checkpoint inhibitors efficacy has been raised. Because antibiotics and antiacids can unbalance gut microbiome, some papers suggested their negative role during immune checkpoint inhibitors treatment (Carbone et al., 2019). However, these data are based mostly on retrospective study and should be addressed on prospective studies focused on this hypothesis. For example, a study in 2022 using a shotgun-metagenomics-based microbiome profiling in a large cohort of patients with advanced NSCLC demonstrated that intestinal Akkermansia muciniphila can predicts increased objective response to immune checkpoint inhibitors treatments (Derosa et al., 2022).

#### 3.2.3 Impact of the use of CAM on efficacy of anticancer treatment

The use of CAM has probably been underestimated in all studies, but 30%–50% of patients with lung cancer probably use them (Wyatt et al., 1999; Micke et al., 2010), which is a higher proportion than for other cancer patients (Johnson et al., 2018). CAM must be thoroughly researched by the oncologist before (and during) the prescription of any treatment, particularly for TKIs. The use of CAM could lead either to side-effects or decrease activity of the antitumor treatment (Berretta et al., 2017). For example, a pharmacokinetic drug-drug interaction was reported between crizotinib and ginger in a NSCLC patient, leading to higher plasma concentrations of crizotinib (via inhibition of the cytochrome P450 3A4 isoform, which metabolizes crizotinib), associated with hepatic cytolysis and discontinuation of the treatment (Revol et al., 2020).

#### 3.2.4 Impact of comorbidity-related medications on adverse drug reactions

Concomitant medication is a risk factor for adverse drug reactions (Hanlon et al., 2006). Most studies have estimated a risk of adverse drug reactions, for which the search is inherent in the process of the pharmaceutical analysis of prescriptions. Taking five or more drugs and an age of over 76 years have been identified as potential factors associated with moderate/severe potential drug related problems (defined as drug interactions, additive toxicity, contraindications) (Puts et al., 2009; Goh et al., 2018). In 244 patients receiving chemotherapy taking a mean of 12 medications (Popa et al., 2014b), the risk of severe non-hematological toxicity was almost doubled for each additional potential drug-drug interaction and tripled for each additional potential drug-drug interaction involving chemotherapeutics. No association between potential drug-drug interaction and hematological toxicity was found. Goh et al., 2018 also reported that PP leading to drug problems was frequent in elderly cancer patients receiving chemotherapy. Another study did not report any association between chemotherapy related toxicity and a higher number of medications (Maggiore et al., 2014). Interestingly, high concomitant medication does not predict adverse radiotherapy outcomes (Nieder et al., 2017).

CAM can lead to antitumor treatment adverse events through pharmacodynamic interactions (Revol et al., 2020), as discussed in Section 1.3.

#### 3.2.5 Impact of comorbidity-related medications on adherence and continuation of anticancer treatments

It is commonly accepted that concomitant medication increases the rate of non-adherence (Gray et al., 2001; Mansur et al., 2009). There is much less data available for lung cancer patients than for breast cancer patients and oral therapy (He et al., 2015) and they do not report non-adherence. In 62 patients treated with erlotinib taking a mean of five co-medications (range from 1 to 13), no association was reported between incorrect intake of erlotinib under fasting conditions and the number of drugs (Timmers et al., 2015). Similarly, Sud et al., 2015 did not identify any association between PP (defined as ≥ 6 medications) and toxicity-related discontinuation of chemotherapy.

#### 3.2.6 Impact of comorbidity-related medications on utilization of healthcare resources

As medications can affect cancer and patient characteristics, they may lead to more utilization of healthcare resources. Taking a large number of medications has been shown to be associated with an increased risk of hospitalizations or emergency room visits (Sud et al., 2015; Hong et al., 2019). Among 298 unplanned admissions of cancer patients, 11% were considered to be associated with an adverse drug reaction and 2% with a drug-drug interaction (Miranda et al., 2011). The most common drug-drug interactions involved warfarin, captopril, and anti-inflammatory agents, and the most frequent adverse drug reaction was neutropenic fever post-chemotherapy. Having a prescription of two or more psychotropic medication classes for at least 90 days in the first year after cancer diagnosis was reported to be associated with a higher number of outpatient visits, office visits, hospitalizations, and longer length of stay (Pamoukdjian et al., 2017). However, these results are controversial (Maggiore et al., 2014).

#### 3.2.7 Impact of comorbidity-related medications on general condition and survival

Medication can be associated with frailty (poor ECOG-performance status) or disability, which can have consequences on the administration and tolerance of antitumor treatment. In a cohort of 290 cancer patients, multiple medication was a risk factor of disability (Pamoukdjian et al., 2017). This finding was supported by another study in which multiple medication was associated with pre-frailty or frailty and poor physical function (Turner et al., 2014). A cut-off between 6 and 9 medications was proposed for predicting the risk of frailty (Williams et al., 2015; Turner et al., 2016).

A recent meta-analysis of 47 studies (not focused on cancer patients) showed a significant association between PP and death. This association increased with the number of medications (Leelakanok et al., 2017). In 289 cancer patients receiving palliative radiotherapy, neither PP nor the use of corticosteroids or opioid analgesics independently influenced overall survival (Nieder et al., 2017). Although potential drug-drug interactions were significantly associated with lower overall survival in breast cancer, this was not true for NSCLC. CAM use without anticancer treatment was independently associated with the risk of death in cancer patients (Johnson et al., 2018).

### 3.3 Impact of lung cancer features on comorbidity-related medications

Although the impact of comorbidity-related medications on lung cancer is described in the literature, there are no data on the impact of lung cancer on concomitant medications. This raises the question of whether the treatment needs to be simplified to avoid known and unknown drug-drug interactions to improve therapeutic adhesion with respect to the clinical benefit when the predicted survival is short. Medication should be re-evaluated on a case-by-case basis according to the type of tumor, its treatment, and the prognosis of the patient. These issues need to be addressed in large-scale studies.

## 4 Conclusion

Lung cancer is the cancer associated with the most comorbidities. These comorbidities and their related medications should be considered in lung cancer management, particularly in the choice of anticancer treatment. There are close relationships between these three entities. They are all intertwined and dependent on each other in lung cancer. Although several observational studies have been carried out, the impact of comorbidity-related medications on the care of cancer patients is less known. Further studies should assess the impact of both medications and comorbidities on lung cancer management and the prognosis of patients. There is a true need in the era of personalized medicine to better understand the impact of comorbidities on cancer and which drugs need to be avoided to optimize patient care.

## Abbreviations

ALK, Anaplastic lymphoma kinase; CAM, Complementary and alternative medicines; CCI, Charlson comorbidity index; COPD, Chronic obstructive pulmonary disease; CTLA-4, Cytotoxic T-lymphocyte-associated protein 4; EGFR, Epidermal growth factor receptor; NSCLC, Non-small cell lung cancer; PD-1, Programmed cell death 1; PD-L1, Programmed death-ligand 1; PP, Polypharmacy; SCLC, Small-cell lung cancer; TKI, Tyrosine kinase inhibitor.
